# Obstetric Surgery1Read at the annual meeting of the Maryland State Homeopathic Medical Society held at Baltimore, October 23, 1907.

**Published:** 1908-08

**Authors:** Dewitt G. Wilcox

**Affiliations:** Buffalo, N. Y., Surgeon to Buffalo Homeopathic Hospital; Gynecologist to Erie County Hospital; Surgeon-in-Chief Lexington Heights Hospital


### Obstetric Surgery.[1]

By DEWITT G. WILCOX, M. D., Buffalo, N. Y.,

Surgeon to Buffalo Homeopathic Hospital; Gynecologist to Erie County Hospital; Surgeon-in-Chief Lexington Heights Hospital.

ONCE eliminate the preventable defects consequent upon the parturient stage, plus the diseases resulting from gonorrheal infection, and the gynecologist would be legislated almost out of office. No man should practise obstetrics, who is not enough of a surgeon to meet the ordinary complications which a minimum number of obstetrical cases is bound to present. Not that every man should be able to make a Cesarean section, but every man should possess enough manual dexterity to enable him to leave the parturient woman in such a state of physical perfection, that she would not in a few years be a neurasthenic or victim of the operating table.

The writer is prone to believe that the average obstetrician of the smaller communities does not fully appreciate what percent of his parturient patients eventually become victims of the operating table. He is, in too many instances, content to point with pride to his brilliant record of ten or more years of obstetrical practice without a death, forgetting that nct alone is he responsible for the mother's life, but in a large measure is responsible for the future good health of that mother, so far as her pelvic organs are concerned. The most frequently observed defects consequent upon the parturient stage, are the lacerated perineum and cervix.

There are instances where a delay of twenty-four hours, or even a week, in repairing the perineum, is not only admissible, but essential to success; but probably in 90 per cent. of instances, immediate repair can and should be made; nor should this repair be done hurriedly and without painstaking effort, for a half healing is but little better than no healing at all and means a subsequent operation. The careful adjustment of each separate muscle and fascia to its fellow of the opposite side, is imperative if good results are to be obtained.

While it is to be assumed that every obstetrician goes to his parturient patient fully prepared to repair a torn perineum,—and anything less than that renders the attendant censurable,—yet there are many instances where through lack of competent assistance, inadequate light, an exhausted patient, and the great tumefaction of the soft parts, due perhaps to a prolonged forceps delivery, that a delay of a day or two may be the means

---

1. Read at the annual meeting of the Maryland State Homeopathic Medical Society held at Baltimore, October 23, 1907.

of securing better results. But before deciding to postpone the repair, he should thoroughly satisfy himself that there is no bleeding from any sizable artery at the seat of rupture. A few buried sutures of ten-day iodised catgut will suffice in bringing together subcutaneously the ends of the separated muscles; then two or more silkworm-gut sutures passed through and through will hold the entire perineum firm and steady. Under no circumstances should silk be used as suture material, as it readily absorbs the infection from the uterine discharge and carries such infection into the deeper layers of the wound.

The after-care of a repaired perineum, whether it be recent or old, is a matter of prime importance. Upon the nurse or attendant there devolves the responsibility of keeping the perineal wound dry and clean. The uterine discharges from a recently emptied womb are quite likely to infect the wound and retard healing. This can be avoided only by changing the dressings frequently, keeping the wound dry and aseptic, and the parts as free from motion as possible. The oft repeated statements made by our older obstetricians, that they have practised obstetrics, ten, fifteen, or twenty years, without having a ruptured perineum, is an acknowledgment of stupidity, for it proclaims an inability to recognise such a condition when existing. In a certain per cent. of cases, a torn perineum is bound to occur, no matter how much skill is employed to avert it, and those cases are so evenly distributed over the country that it is scarcely possible one man will be so fortunate as to escape finding one in twenty years practice.

The immediate repair of a lacerated cervix is an open question. The weight of opinion is overwhelmingly against such a procedure. We have, in the cervix, a different structure to deal with, as well as one differently located; ordinary laceration tends to heal spontaneously, and those so badly torn that they fail thus to heal, would probably not heal if sewed. The lips are tumefied, the structure soft, and a ligature, especially if tied tightly at first, would soon become loose after the tumefaction subsided. While a late operation is best for repairing the cervix, yet it should not be postponed longer than is necessary for the woman to gain strength and be in a condition for such an ordeal. The sooner it is done, the less liable is she to suffer reflex irritability. Three months should be the maximum time allowed before repairing a lacerated cervix. There is one condition, however, which calls for immediate repair of the cervix, and that is profuse bleeding from the circular artery or possibly the uterine artery. When such is the case, it is essential to the safety of the mother, that the cervix be repaired immediately.

Next in order comes subinvolution, that forerunner of uterine displacements, pelvic congestion, and general pelvic distress. Days or weeks are not the true index governing the lying-in period of the parturient woman, but rather the process of involution. What may be a long period to one woman becomes short to the next, simply because her uterus has not had sufficient time to regain its normal size. No better foundation could possibly be laid for a future operation than to allow a parturient woman to get out of bed, go about her duties, indulge in marital relations and otherwise take her place in the family, when the uterus is still large, flabby and filled with unabsorbed and hyperplastic connective tissue. Rare indeed, would be the instance wherein such a woman did not eventually find herself seeking the assistance of the gynecologist to aid her in overcoming a retrodisplacement, prolapse, procidentia, or chronic endometritis.

Not infrequently does the opportunity come to the obstetrician, to cure at the time of his patient's confinement, a previously existing retrodisplacement. A uterus which has been so displaced, and is large and flabby, becomes after confinement a very tractable uterus, freely amenable to a line of treatment which was not possible before confinement, and which may forever cure the patient of her difficulty, and possibly avert a future operation.

Naturally, all the adhesive bands have been broken up by the gestative process. Naturally, also, has the overabundant connective tissue been absorbed and the overdistended bloodvessels become pliable and ready to contract. By the thorough removal of all membranes and shreds from the uterine cavity, by keeping the patient off her feet longer than the usual time after her confinement, by the employment of frequent hot douches, by the judicious use of tampons, the knee-chest position, having the patient lie on her stomach rather than upon her back, and by continuing the nursing of her child at the breast, such a condition as chronic subinvolution or displacement can be cured at the time of confinement.

The discovery of the cause of the various infective diseases of the fallopian tubes, ovaries and uterus, such as pyosalpinx, infective ovaritis, endometritis, salpingitis, and the like, has opened our eyes to the great risk which the parturient woman runs of becoming a chronic invalid, and later a victim of the operating table, through puerperal infection of the uterus and the uterine adnexia. It would seem almost like arrogance on my part to suggest the necessity of strict asepsis in the practice of midwifery, as that is a recognised and undisputed duty of the obstetrician. If medical science has demonstrated anything to our entire satisfaction during the last decade, she has forced us to a recognition of the power of bacterial invasion through the medium of the uterine mucosa. Yet the indifference to this truth, displayed by a certain few of the obstetricians, even today, results in no small percentage of their patients eventually requiring an abdominal section for the removal of infected tubes or pelvic adhesions, which have rendered said patients semi-invalids for a period of years.

Experience and observance in pelvic work have convinced the writer that no woman can suffer an attack of puerperal fever of average severity, without being left permanently damaged in some of her pelvic organs. The facts that she may recover from the immediate attack, that she may be able to bear more children, that she is apparently able to perform her regular duties, are not sufficient arguments to sustain the contention that she has not been so damaged. As the mills of gods grind slowly, so does the latent infection consequent upon puerperal fever develop slowly but exceeding sure. After the initial attack the patient may appear to be well and years may elapse ere the trouble shows itself, when suddenly, after a severe cold, an overstrain, a miscarriage or the advent of the menopause, there develops alarming symptoms, which only an abdominal section can assuage. Then is laid bare the whole story from the beginning, with its startling moral—inflamed adherent tubes; chronic pelvic peritonitis; a retrodisplaced uterus with unyielding bands of adhesions or a tubo-ovarian abscess, all or any one of which was the result of the latent infection planted years ago at the parturient period, and all of which might have been averted by a recognition of, or a careful attention to the unfailing law of bacterial invasion through the uterine mucosa, at the time of confinement.

There are a few operations, which must occasionally be performed during or at the close of the pregnant stage. First, is that delicate procedure which, while it is but rarely demanded, is nevertheless of sufficient importance to entitle it to careful attention; but strange to say, it has received but scant consideration from the men who are best qualified to speak upon it. It is the induction of abortion and miscarriage.

It is but natural and therefore commendable that the honest doctor should shrink from anything pertaining to the premature emptying of the uterus. The honest policeman shrinks from taking a human life, even though it be to save another, yet he must occasionally do it in the discharge of his duties. So the physician, who is the policeman against disease and death, must at times sacrifice one life to save another apparently more valuable. In rare instances, such as a dead fetus, an intractable, adherent, retroverted uterus, a placenta previa, a fibroid tumor, cervical cancer, persistent and exhausting vomiting, dementia, Bright's disease, or any condition which seriously threatens the life of the mother, it becomes absolutely essential to the preservation of the mother's life that the uterus be emptied before full term, It is here that our literature is scant as to the best method of procedure. Nature jealously guards her offspring. To induce a uterus to part with its incompleted product, is a task frequently difficult and frought with grave peril. While nature may herself, as in the case of a dead fetus, recognise the difficulty and seek to expel it, yet her tardiness in the recognition and expulsion may cost the life of the mother; hence the physician must have at hand some reliable method of recognising the danger, and some prompt, efficient and safe method of emptying the uterus in just such unfortunate circumstances.

Given a patient who is afflicted with some of the above conditions, wherein a consultation of two or more physicians results in a verdict of abortion, what is the first move? Obviously, the selection of the time must be determined first. Early writers tell us it is better to empty the uterus at what would be the menstrual period, as the tendency to uterine contraction and placental expulsion is then greater. That argument is valid only in case we intend to induce artificial labor and not empty the uterus by force. If there is the possibility of delivering a viable child, then we may wish to induce artificial labor and the time of the month is a consideration; but with no prospect of a viable child, there is little necessity of paying any attention to the would be menstrual periods. The introduction into the uterus of foreign bodies, such as sounds, tents, gauze, catheters and the like, to induce labor, belongs to the antiquated period of septic carrying days, and is not to be considered as an excusable method, save perhaps in the first few days of pregnancy.

If an abortion is to be induced, and in using that word the writer means the emptying of the uterus at or before the fourth month there is just one safe, rapid and effectual method of doing it, and that is this: first, a thorough bowel evacuation; second, cleansing the vagina and vulva, with shaving and douching; third, a general anesthetic, or possibly the injection of cocaine into the cervix. Then place the patient in the lithotomy position, with a good light. Sterile hands and instruments are of course essential. Retracting the perineum, by means of retractors, brings the cervix into view. This is seized with tenaculum forceps and held steady while graduated sounds are introduced one after another into the uterine cavity. A point of prime importance in the first introduction of a sound or dilator into the uterine cavity, is to know the position of the fundus. Failure to know this fact has resulted not infrequently in perforation of the uterine wall.

The introduction of a sound may or may not be an easy process, depending upon the rigidity of the os and the extent to which it must be dilated. The shorter the pregnancy, the more rigid the os, as a rule. I have seen cases, where it required one hour of the hardest kind of work to dilate the os sufficiently to deliver a four months fetus. After the sounds have done their work, the Goodell dilator comes next. If the thumbs can be employed after the dilators are of no further use, they no doubt afford the safest means of accomplishing the end, but frequently the os is so rigid that but little can be accomplished by that method. The free use of sterile oil or vaseline will facilitate materially the process of dilatation. The method of introducing one finger after another, by making a wedge of them, is very satisfactory. In the absence of any other instrument, I have found a Pratt rectal speculum as satisfactory as anything with which to complete dilatation. Recently my attention was called to the Newell dilator, which I have found extremely satisfactory. It is less likely to tear the surface of the os and dilates very uniformly.

When dilatation is sufficiently complete to deliver the contents, the size of the fetus determines the next step. If it is three months old or over, some form of instrument must be used to seize the head of the fetus and deliver it in toto; otherwise it is torn to pieces and the danger of infection, lacerations and incomplete delivery is present. To meet this requirement the writer has had made a pair of miniature obstetrical forceps, just large enough to seize a three or four months fetus and deliver it without tearing. The lack of such an instrument has caused him, upon a few occasions, more annoyance and vexation than the complications of many a major operation.

After the delivery of the fetus but little difficulty will be encountered with the placenta if it is seized with placental forceps and carefully delivered. A brisk hemorrhage is likely to follow the placental delivery, but it is quickly controlled by the next step, which is a thorough curettage of the entire endometrium, to insure the removal of all shreds and a firm uterine contraction. The douche curet is ideal for this part of the work. No pains should be spared to free the uterus of all membranes. It is not a question of forcibly scraping the uterus, for that is unnecessary and harmful; but rather of reaching the entire surface, especially the two corners of the uterus. The use of strong chemicals in the douche water is unnecessary; salt or plain sterile water is quite sufficient. I make a practice of packing and draining every uterus thus delivered. The iodoform tape, one-half inch wide, with edges folded in and long enough to fill the uterine cavity, is sure to absorb and antidote all possible infection that may be gathered there. This can be removed in twenty-four or forty-eight hours, according to requirements. A rest in bed, commensurate with uterine involution, is the only guide for convalescence.

The mortality attending such procedure is practically nil. If infection has taken place and becomes well established before the operation, then perforce it may continue to a fatal end in spite of the interference. In placenta previa, the management is much the same, only the work must be done with dexterity and despatch, to avoid the bleeding so generally present in such cases. The writer is of the opinion that it is better in such cases to empty the uterus forcibly and quickly, as above described, rather than to induce labor and wait for nature to expel the contents. The only modification to be made in the procedure, in the case of placenta previa, is not to introduce the sound or dilator further than within the internal os. After dilatation is sufficiently complete, the finger can be slipped alongside the placenta and detach it from the uterine mucosa. Its delivery then is a very simple matter.

In pregnancy complicated by fibroid tumors, much has been written. I have seen comparatively few cases in which the tumors were so situated as to interfere with normal labor; but I have seen many cases, where the patients had one or more fibroids, which did not in the least interfere with the parturient process. It is not always necessary to abort a woman who has a fibroid so situated as apparently to act as a bar to delivery. It is possible frequently to remove the tumor by abdominal section during the gestative stage, and thus best serve the interest of mother and child. It is surprising and gratifying to observe the surgical ordeals, through which a pregnant woman will pass, without miscarrying.

A paper on the subject of "Obstetric Surgery" would not be complete without at least a few words on that peculiar anomaly of pregnancy, ectopic gestation. It is a well known physiological fact that the uterus undergoes a certain preparation for the reception of the ovum each month. A few days prior to the arrival of the expected guest, that organ cleans house, as it were, and adds new interior furnishings; old shreds of membrane are cast off and a new heavy lining, of a velvety character, richly supplied with blood connections, is formed in the upper part of the uterine cavity: this is the decidua. If the ovum arrives as guest in ordinary (that is, unimpregnated) then the new furnishings are torn down and cast off, and form part of the menstrual flow. If, however, the ovum comes as a royal guest, (impregnated and developed up to six or eight days) then is the reception made fitting to the ovum. The doors and windows of the decidua are closed, to prevent the untimely escape of the ovum, or intrusions from without. Richer blood supply is added and the velvety hangings are materially increased in thickness. One corner of the reception hall is set aside for the guest, where he is permanently stationed and sumptuously fed. This guest, the ovum, sends out rootlets or villous growths, which ramify into the decidua and the circulation between uterus and ovum becomes established.

It becomes a most entertaining sight to the scientist to watch the conduct of affairs when the host (uterus) has been assured that a royal guest will soon arrive and the latter fails to keep his appointment. The ovum, impregnated in the tube in the usual way, sends couriers ahead, announcing its early arrival in the uterus. That organ makes immediate preparations to receive the guest. The eight days expire but the guest has not appeared; ten days and two weeks, yet he fails to come. The furnishings and hangings are wrought more elaborately, as though that would hasten him. The uterine decidua has attained two or three times its normal size, but at the end of four, or perhaps five weeks, no guest has appeared, and in disgust at such uncivil treatment, the host ruthlessly tears out all evidence of the elaborate preparations and casts them forth. The decidua has been expelled, and here note that we have in this act, one of the most positive signs of tubal pregnancy. This decidua may be the size of a placenta in a three months uterine pregnancy, and the physician, if he is not alert, will be induced to think he is dealing with an ordinary abortion and may search long and carefully for a fetus, which comes not.

We now go back to the ovum and see what sort of treatment it has received at the hands of the tube, which has aspired to the dignity of entertaining the royal guest, who had intended making there only a short sojourn on the road. This tube, recognising that it must prepare a fitting chamber for one of honor has, upon short notice, added new furnishings, which heretofore were foreign to so humble a house. A tubal decidua has formed about the ovum, which has increased in size as the ovum grew, until at the end of two or three weeks, she is able to provide her guest with all the nourishment and necessities that the royal palace of the uterus could provide, only in cramped quarters. But the ambition of the tube has not reckoned with her capacity or powers of endurance, for the royal guest has so increased in size that the walls of the house are full to bursting, and impending disaster threatens.

A scarce never failing sign of ectopic pregnancy is an impression which the patient has that she is pregnant. Particularly is this present if she has borne children. She will state that she has a peculiar, indescribable sensation in the pelvis; the abdomen is slightly sensitive; there is a pain in ovary or tube upon bowel movements; there is some increased frequency of urination. She may or may not feel nauseated, but is likely to feel so after four or five weeks have elapsed. The time comes for her to be unwell, but no flow appears. If her habit be that of prompt regularity, this is almost a positive sign of pregnancy, but not necessarily of tubal pregnancy. In two or three days her anxiety is relieved by discovering a little flow, but this ceases perhaps after six, twelve, or twenty-four hours. This flow is caused by the uterus in its attempts to get rid of the unnecessary decidua for, be it remembered, that the ovum now impregnated in the tube, may have been lodged there four weeks previous, at the last menstrual period, and at the present moment, although she has missed but one period by a few days, she may have an ovum of four weeks development in the tube; hence, quite a decidua has formed in the uterus, which that organ is endeavoring to cast off. This periodic irregular flow of blood is an important sign.

Most of the above symptoms give the physician a suggestion that the patient is pregnant normally, but this show of blood a week or so after the period is due, should arouse his suspicions that all is not right. He need not wait long for a confirmation of these suspicions; it will come if he is watchful. Next, he is told that there is more flow, which, like the other, lasts a few hours and then ceases, or may appear only when the patient is on her feet. She complains of an unpleasant sensation in the right or left ovary; it has become more sensitive, she avoids sudden movements or jars. About this time the patient may have sudden severe sharp pains in the sensitive side; these may be of a character to make her scream out in agony or fall in a faint on the floor. These pains may be repeated in a few hours or days, or may only come when she makes an exertion, but with each pain she is quite likely to have a little uterine flow. Now it is not to be understood that this pain means a rupture of a tube, because one tube cannot rupture every two or three hours or days; but it does mean that the tube has become as full as it will hold with the constantly increasing ovum as a guest, and because it cannot further stand such internal pressure, its peritoneal covering has begun to tear in places and the consequent pain results. Each one of these tears seem to stimulate the uterus to contract, hence we have an external show of blood.

If a physician sat at the bedside of a patient, whom he had never before seen. and she related to him such symptoms as above mentioned, he could not be excused for failing to recognise the true condition; but if he were in doubt he has the means at hand for satisfying himself beyond all question, and these are: the discovery, by a bimanual examination, of an enlargement to the right or left of the uterus. This may be as large as a walnut, a lemon, an orange, or it may be balogna shaped. It is extremely sensitive and must be handled carefully. It will also be made known by the examination, that the uterus is enlarged almost to the extent of the duration of the suspected pregnancy. The cervix will be soft and will be purple in color like a normal pregnancy. A few days later the physician may be told by the patient that her troubles are over, as everything has come away, and she shows him what appears to be an afterbirth. It is so nearly like it that he may not detect a difference, but his precaution will induce him to make an examination. He finds the so-called placenta is, in fact, a thin membrane, rather tent-shaped, which he can slip over his finger, and that it has not the characteristics of a placenta. He finds that while the uterus is smaller than before, there is still the same lump in the side, just as sensitive, and she still has her symptoms of pregnancy. It was the decidua which has come away and not a placenta, due to an abortion. Moreover, she will still continue to have her agonising pains, after each one of which she feels faint. She shows clearly the loss of blood; her lips are pale; her eyes hollow and she is constantly thirsty.

We will now suppose a period of from six weeks to two months has elapsed since the suspected pregnancy began. An urgent call reaches the physician to come to his patient immediately; she is dying. He finds her pulseless, blanched, breathing softly, cold and clammy. A friend says, "She screamed out with agony, put both hands over her lower abdomen, tried to get up and fell back fainting." We have now come face to face with an issue which nature has been predicting so forcibly, if we could but understand her language.

The tube has ruptured from end to end, or such portion of it as contained the fetus. The thousands of little rootlets, which carried blood from the lining of the tube to the tubal decidua, have been torn open and are pouring quantities of blood into the abdominal cavity; or the ovarian artery may have been ruptured also, and thus is the hemorrhage increased. Even yet the patient is alive, hence there is hope of saving her, and it is far better to recognise the true condition and apply the remedy, even at so late an hour, than to allow it to go unrecognised and death ensue.

It need not be said that the one remedy is a rapid abdominal section, securing bloodvessels, turning out clots, getting the patient back to bed as quickly as possible and giving normal saline enemata or intravenous injections, together with all other means for combatting shock. But how infinitely better to have determined the question earlier and applied a similar remedy with one hundred chances in favor of success.

Were I to detail all operations complicating pregnancy, I should prolong my paper far beyond the time limit. Cesarean section, either abdominal or vaginal, is so rarely demanded, that it is interesting to the surgeon only; yet the mortality of this procedure is now so low and the results to mother and child so gratifying, that it should always be considered before determining upon a premature delivery, because of a deformed pelvis.

Symphyseotomy has a logical place in difficult or impossible deliveries, and can be performed quickly and safely.

The one thought of the paper is that the obstetrician has it in his power to so conduct all the details of his obstetrical practice that but a minimum number of his patients need ever become victims of the operating table, or neurasthenics.

173 Lexington Avenue.

